# Expression of the Bcl-2 Protein BAD Promotes Prostate Cancer Growth

**DOI:** 10.1371/journal.pone.0006224

**Published:** 2009-07-13

**Authors:** Adrienne J. Smith, Yelena Karpova, Ralph D'Agostino, Mark Willingham, George Kulik

**Affiliations:** 1 Department of Cancer Biology, Wake Forest University School of Medicine, Winston-Salem, North Carolina, United States of America; 2 Comprehensive Cancer Center, Wake Forest University School of Medicine, Winston-Salem, North Carolina, United States of America; 3 Department of Pathology, Wake Forest University School of Medicine, Winston-Salem, North Carolina, United States of America; Health Canada, Canada

## Abstract

BAD, a pro-apoptotic protein of the Bcl-2 family, has recently been identified as an integrator of several anti-apoptotic signaling pathways in prostate cancer cells. Thus, activation of EGFR, GPCRs or PI3K pathway leads to BAD phosphorylation and inhibition of apoptosis. Increased levels of BAD in prostate carcinomas have also been reported. It appears contradictory that instead of limiting expression of pro-apoptotic protein, prostate cancer cells choose to increase BAD levels while keeping it under tight phosphorylation control. Analysis of the effect of BAD on prostate cancer xenografts has shown that increased BAD expression enhances tumor growth, while knockdown of BAD expression by shRNA inhibits tumor growth. Tissue culture experiments demonstrated that increased BAD expression stimulates proliferation of prostate cancer cells. These results suggest that increased expression of BAD provides a proliferative advantage to prostate tumors, while BAD dephosphorylation increases sensitivity of prostate cancer cells to apoptosis. Combination of proliferative and apoptotic properties prompts prostate cancer cells to be “addicted” to increased levels of phosphorylated BAD. Thus, kinases that phosphorylate BAD are plausible therapeutic targets; while monitoring BAD phosphorylation could be used to predict tumor response to treatments.

## Introduction

Prostate cancer is the most frequently diagnosed cancer and the second leading cause of cancer-related deaths in men in the United States [1]. Currently there is no effective treatment for androgen-independent advanced prostate cancer [2]. Mechanisms that enable prostate cancer cells to evade apoptosis may contribute to therapeutic resistance. Thus, increased levels of several growth factors, including FGF, EGF, IL-6 and GPCR agonists that activate anti-apoptotic signaling pathways, have been reported in androgen-independent prostate cancer [3]–[7]. Anti-apoptotic signals could either post-translationally modify apoptosis regulatory proteins or change their expression levels. Indeed, increased expression of anti-apoptotic Bcl-2 proteins as well as inhibitors of apoptosis proteins (IAPs) in advanced prostate cancer has been reported [8], [9]. Also, we have recently shown that in prostate cancer cells, the pro-apoptotic Bcl-2 protein BAD plays a unique role as a convergence point of several anti-apoptotic signaling pathways that include constitutively active PI3K, activated EGFR and GPCR [6].

BAD, **b**cl-xl/bcl-2- **a**ntagonist causing cell **d**eath, was initially identified in a yeast two hybrid screen interacting with Bcl-2 or Bcl-xl [10]. BAD is a unique BH3-only family member in that its regulation is primarily mediated through its conserved phosphorylation sites (serines 112, 136, and 155 based on the mouse sequence)[11], [12]. Phosphorylated BAD fails to bind Bcl-XL or Bcl-2 proteins, and has been considered an apoptosis sentinel inactivated by anti-apoptotic signals. Upon withdrawal of survival factors BAD becomes dephosphorylated, shifts the balance of pro- and anti-apoptotic Bcl proteins that triggers release of cytochrome c, SMAC and AIF from mitochondria and subsequently leads to apoptosis [12]. Thereby, it would not be surprising if cancer cells decrease BAD expression.

A recent study has shown that BAD expression is elevated in prostatic carcinomas compared to low expression in normal prostatic epithelium [13]. It seems counterintuitive that prostate cells would dedicate extra resources to maintain BAD phosphorylation instead of eliminating its expression. It is possible that in addition to regulating apoptosis, BAD might play a positive role in prostatic tumor growth.

Here we report that increased BAD expression stimulates proliferation of prostate cancer cells in tissue culture and prostate tumor growth *in vivo*. At the same time, BAD dephosphorylation increases sensitivity of prostate cancer cells to apoptosis. This combination of proliferative and apoptotic properties creates conditions for prostate cancer cells “addiction” to increased levels of phosphorylated BAD. Thus, kinases that phosphorylate BAD are plausible therapeutic targets.

## Materials and Methods

### Cell lines

Prostate cancer cell lines, LNCaP and C4-2, were gifts from Dr. Leland Chung (Emory University, Atlanta GA). C4-2BADLuc cells were generated by transfecting C4-2 cells with wild-type BAD (HA-BAD-pTRE2hygro) and firefly luciferase (PGL3) whereas pTRE2hygro and firefly luciferase (PGL3) were transfected into C4-2 cells to generate C4-2Luc. LNCaP cells were maintained with T-medium supplemented with 5% fetal bovine serum, and C4-2 cells were maintained with RPMI 1640 with 10% fetal bovine serum. All cells were kept at 5% CO_2_ at 37°C.

### Antibodies and Other Reagents

Antibodies were obtained from the following sources: BAD, phospho-specific BAD (serines 112, 136, 155) from Cell Signaling Technology (Beverly, MA); ERK from Zymed Laboratories (South San Francisco, CA); secondary horseradish peroxidase-conjugated antibodies used for Western blots from Amersham Biosciences (Piscataway, NJ). All other chemicals (unless specified) were purchased from Sigma (St. Louis, MO). Tissue culture reagents were purchased from Invitrogen (Carlsbad, CA).

### shRNA experiments

A lentiviral vector (pLL3.7) [14] was used with an shRNA insert of annealed oligonucleotides. The BAD DNA target sequences used were 5′-TGAAGGGACTTCCTCGCCCGT-3′ and 5′ GGCTTGGTCCCATCGGAAG-3′. HEK 293 cells were transfected with pLL3.7 vector containing either of these sequences or a scrambled sequence 5′-GGTACGGTCAGGCAGCTTCT-3′ in combination with packaging vectors (VSVG, RSV-REV, and pMDL g/p RRE). After 48 h, supernatants were collected from these cells and used to infect LNCaP or C4-2 cells [6]. Forty-eight hours after infection, cells were plated for subsequent experiments.

### Proliferation Assays

Cell counts were done by the following: 2×10^5^ cells were plated in six cm dishes for each experimental group. The initial cell count was 24 hours after cells had attached to the dishes (Day 1). Two additional counts were made three days later (Day 4) and six days later (Day 7) and then compared to the initial cell count. Counts were made by trypsinizing and collecting cells in media, then manually counting on a hemacytometer. MTT assays were done according to instructions of kit manufacturer (Roche Applied Science, Indianapolis, IN) on cells plated in 24 –well plates at varying densities. Triplicate wells were used for each data point.

### Immunohistochemistry

Antibody staining was performed on histological sections of formalin-fixed prostate tumor xenografts. Antigen retrieval was performed by heating slides at 95°C in 10 mM sodium citrate buffer (pH 6.0) for 60 min. Then, sections were treated identically as follows: 1) incubated in 2% hydrogen peroxide to block endogenous peroxidase activity; 2) incubated with blocking solution: 1% BSA, 0.1% tween20 in PBS (30 min, 25°C); 3) incubated with primary antibodies, Ki-67 from Abcam Inc. (Cambridge, MA) diluted 1∶25–1∶200 in blocking solution (overnight, 4°C); primary antibodies were followed by peroxidase conjugated anti-rabbit secondary antibodies (10 µg/ml, in blocking solution, 30 min, 25°C) and revealed with 3-3′-diaminobenzidine (DAB) as the developing chromogen. Between steps, specimens were washed in PBS 3 times.

### Subcutaneous Implantations

Nude mice (BALB/cAnNCrj-nu from Charles River) received four subcutaneous injections of 2×10^6^ cells with Matrigel. Injections were made using an insulin syringe and a 27 gauge needle. All manipulations with animals were conducted in humane manner, in strict adherence with a protocol approved by institutional ACUC, which was designed to minimize animal suffering.

### Luminescence Imaging

Tumor growth was analyzed with a Xenogen IVIS® 100 optical imaging system (Caliper Life Sciences, Hopkinton, MA). Animals were immobilized for substrate injection and imaging through an attached gas anesthesia system consisting of 2% isoflurane/O_2_. To account for background and nonspecific luminescence, mice were imaged before injection of luciferase. Animals were injected with 100 µl of the firefly luciferase substrate luciferin (3.5 mg/ml in PBS) and imaged 15 minutes later in prone and supine positions (5 minutes each). Whole-body images were obtained using the Living Image® software provided with imaging system. A gray-scale photographic image and the bioluminescent color image are superimposed to provide anatomic registration of the light signal. A region of interest (ROI) was manually selected over the luminescent signal, and the intensity was recorded as photons/second within an ROI.

### Statistical analysis

To determine whether differences between data sets were statistically significant, Student's t-test analysis (two-tailed distribution; two-sample unequal variance) was performed using Excel software.

## Results

### BAD expression stimulates proliferation of prostate cancer cells

**Figure 1:**
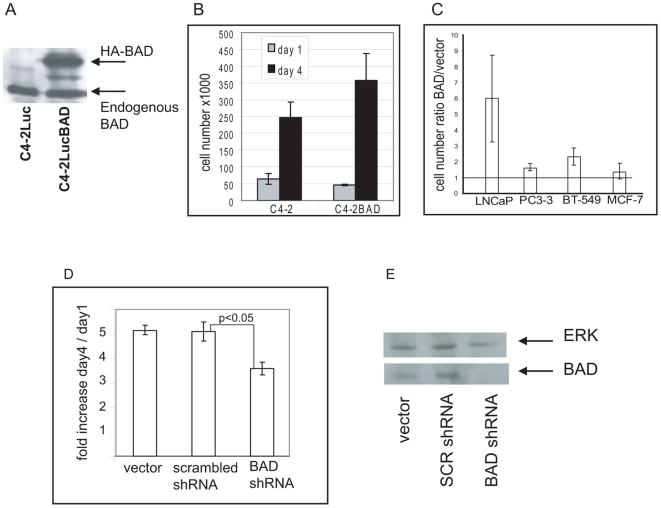
BAD expression increases proliferation of prostate cancer cells. (A) HA-BAD expression in C4-2LucBAD clone. (B) C4-2LucBAD cells proliferate at a faster rate than C42 cells. C4-2LucBAD cells or C4-2Luc cells were plated in triplicate 6 cm dishes. At days 1 and 4 after plating, cells were trypsinized and counted. Results at 4 days were significantly different with a p value of <0.001. Comparable results were obtained in experiments in which proliferation was measured with the MTT assay. C) Transient expression of HA-BAD stimulates proliferation. LNCaP cells were transfected with 9∶1 mixture of GFP and either of HA-BAD or empty expression vector. Seven days after transfection, the number of GFP positive cells was counted. Graph shows the HA-BAD/empty vector ratio of GFP positive cells. Proliferation of GFP-positive cells was confirmed by time lapse video recording. D) Knocking down BAD expression with shRNA decreases proliferation. A lentiviral vector (pLentiLox 3.7) with a BAD shRNA insert was used to infect C42cells. C4-2Luc cells were plated in triplicate 6 cm dishes. At day 1 and 4 after plating, cells were trypsinized and counted. Experiments were repeated at least 3 times. E) Western blot analysis of BAD expression in cells infected with empty lentiviral vector, scrambled shRNA or BAD-specific shRNA. Expression of total ERK was used as loading control.

Reports on increased expression of BAD in prostate cancer led us to suggest that prostate cancer cells may benefit from maintaining BAD expression. To address the possible role of increased BAD expression, we examined proliferation of prostate cancer cells that overexpress BAD. For this purpose, we compared proliferation of cell lines that ectopically express BAD and cell lines transfected with empty vector. Cells with elevated levels of BAD were characterized by increased proliferation in tissue culture (Fig. 1A, B). To exclude the possibility that increased proliferation of cells that stably express BAD was due to clonal variations, we compared proliferation in cells transiently transfected with either BAD or empty vector. These experiments showed increased proliferation in several cell lines that transiently express BAD (Fig. 1C).

Since C4-2 cells are derived from metastatic prostate cancer [15], it is possible that they may have already established optimal levels of BAD. Therefore, we examined the effect of knocking down BAD expression on proliferation of these cells. C4-2Luc cells were infected with lentiviral vectors that encode scrambled shRNA or BAD shRNA. Reduced expression of BAD led to decreased proliferation of C4-2 cells (Fig. 1D, E).

### BAD expression stimulates prostate tumor growth

**Figure 2:**
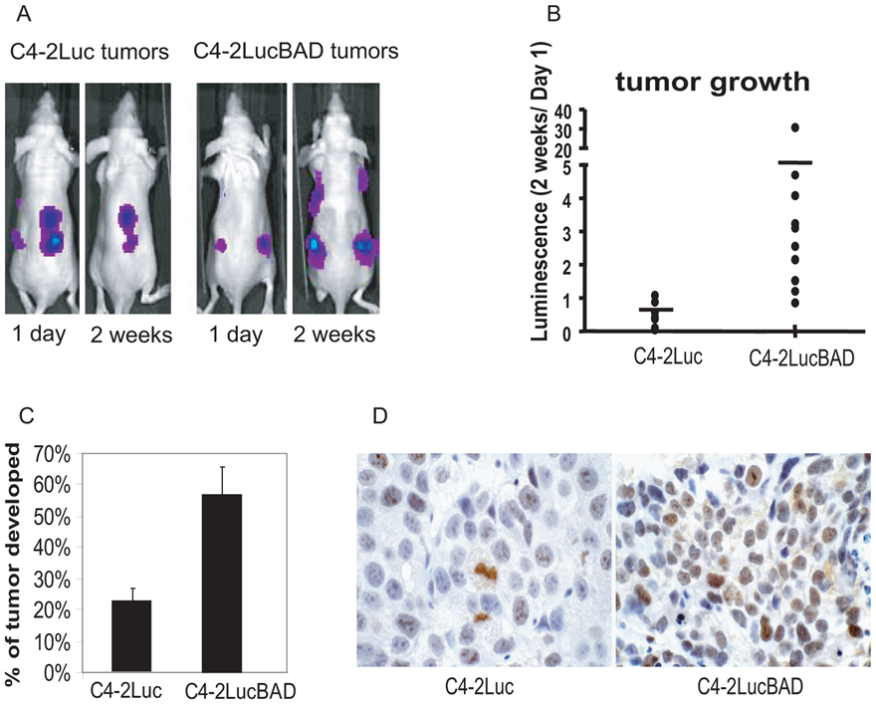
Over-expression of BAD increases tumor growth rate and tumor take. Nude mice received four subcutaneous injections of 2×10^6^ C4-2Luc or C4-2LucBAD cells. A) Representative whole body images of the animals obtained at 1 day and 2 weeks after implantations using the IVIS100 and Living Image® software (Xenogen). B) Dot plot showing fold increase and median luminescence in mice injected with C4-2Luc and C4-2LucBAD cells. C) Percent of palpable tumors (over 5 mm) developed at injection sites. D) Representative tissue sections of formalin-fixed tumors stained for proliferation marker Ki67.

Experiments in tissue culture have shown that expression of BAD stimulates division of prostate cancer cells as well as other cancer cells. To determine whether increased expression levels of BAD stimulate prostate tumor growth *in vivo*, we compared growth of C4-2Luc and C4-2LucBAD cells implanted in immunocompromised mice. C4-2Luc and C4-2LucBAD cells express firefly luciferase that allows the monitoring of xenograft growth noninvasively by optical imaging. Measuring luminescence instead of physical tumor size permits detection of xenograft growth prior to the appearance of palpable subcutaneous tumors. This approach reduces the time required to measure kinetics of tumor growth. Analysis of luminescence of C4-2Luc and C4-2LucBAD xenografts showed increased tumor take and faster tumor growth of C4-2LucBAD xenografts (Fig. 2AB). Consistent with results of luminescence analysis, C4-2LucBAD cells produced palpable tumors at higher frequency comparing to C4-2Luc cells (Fig. 2C). In accordance with the faster growth of C4-2LucBAD xenografts, immunohistochemical analysis showed an increased number of cells that stained positive for the proliferative marker Ki-67 compared to C4-2Luc xenografts (Fig. 2D).

### Knockdown of BAD expression by shRNA inhibits tumor growth

**Figure 3:**
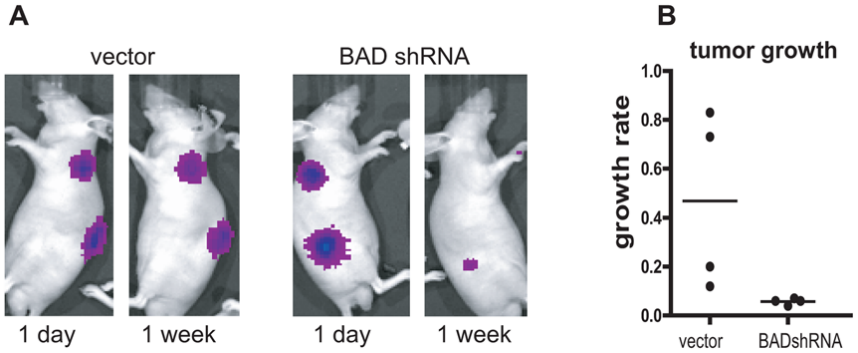
Knocking down of BAD expression with shRNA inhibits growth of prostate cancer xenografts. Nude mice received two subcutaneous injections of 2×10^6^ C4-2Luc cells infected with lentiviral vector with BAD shRNA (right side) or an empty vector (left side). Images and quantification are as in Figure 2. A) Representative images of C4-2Luc and C4-2LucBAD tumors. B) Dot plot shows the ratio between luminescence at one week/day 1. After eight weeks, palpable tumors were detected only at sites injected with C4-2Luc cells infected with empty vector.

Parallel to experiments with C4-2LucBAD cells, experiments were conducted with C4-2Luc cells in which endogenous BAD expression was inhibited by the shRNA approach. C4-2Luc cells were infected with the lentiviral vector pLL3.7 that expressed BAD shRNA or scrambled shRNA, and the cells were then implanted subcutaneously into nude mice. Luminescence of C4-2Luc xenografts was followed for one week as shown in Fig. 3. C4-2Luc xenografts with a reduced expression of BAD showed reduced tumor take and grew at a slower rate than cells with intact BAD expression.

## Discussion

### The novel role of BAD in promoting tumor growth

The results presented in this paper show that BAD, the BH-3 only Bcl-2 protein, might function in a dual capacity in prostate cancer. When dephosphorylated, BAD promotes apoptosis [11], while in a phosphorylated form it stimulates proliferation and tumor growth *in vivo*. This connection between BAD expression and proliferation provides a possible explanation for an increase in expression of phosphorylated BAD protein in prostate tumors.

Recently, several studies have shown that functions of BAD may extend beyond sensitizing cells to apoptosis. For instance, publications from the Peter Vogt and Elizabeth Yang laboratories have suggested that BAD protein can be involved in promoting cell cycle progression [16], [17]. Thus, fibroblasts with increased expression of Bcl-2/BclXL are characterized by reduced apoptosis and also by decreased proliferation. However, when Bcl-2 or BclXL forms a heterodimeric complex with BAD, cells can overcome the G0/G1 growth arrest and enter into S phase [17], [18]. These findings were extended to T cells by showing that T-cells over-expressing BAD were more likely to remain in S-phase [19].

In other recent reports, BAD in the phosphorylated form was found to promote assembly of active glucokinase complexes, an initial step of the glycolytic pathway [20], [21]. Although both increased proliferation and glycolysis are hallmarks of tumor growth, the experimental evidence that connects BAD expression with tumor growth has been lacking.

#### Could BAD play a dual role in prostate cancer cells?

Several reports have shown that cells that express BAD proliferate faster; however, mechanistic details on how BAD promotes proliferation diverge. One possibility is that BAD provides a counterbalance for increased levels of BclXL and Bcl-2 that are known to slow proliferation [22]. If this scenario is correct, any pro-apoptotic Bcl2/BclXL antagonist would be expected to have a BAD-like effect. However, if expression of such an antagonist is constitutive, it would defeat the purpose of increased Bcl-2/BclXL expression by increasing apoptosis sensitivity. Since the proportion of BAD that could form heterodimers with anti-apoptotic counterparts depend on phosphorylation status, BAD may be uniquely suited for the role of modulator of BCl2/BClXL, availability of which is fine tuned by protein kinases. It is also possible that by increasing rate of glucose utilization, BAD expression provides competitive advantage to the cells in hypoxic tumors that switch from oxidative phosphorylation to glycolysis [23].

The precise mechanism of how Bcl proteins regulate proliferation is obscure. It remains to be determined whether a single mechanism plays a dominant role or BAD-dependent stimulation of proliferation is mediated via several mechanisms simultaneously, and whether BAD localization to a specific organelle (e.g. mitochondria, ER, nuclear envelope) is important. Also, this positive effect on cell division may not be uniformly manifested in all cancer cells. Thus, BAD reportedly inhibits G1 to S transition in MCF7 breast cancer cells [24]. Until the effects of BAD on proliferation are dissected on a molecular level, we remain with the notion that effects of BAD expression on proliferation are cell type-dependent.

### Conclusions

Regardless of the exact mechanism that permits BAD to stimulate tumor growth, this capacity may provide selective pressure to increase BAD expression in tumors. Activation of protein kinases that phosphorylate BAD creates a permissive condition to increased expression of BAD. It is tempting to speculate that that high BAD expression should make these tumors increasingly sensitive to inhibitors of signaling pathways that control BAD. If so, high levels of phosphorylated BAD could be used to identify patients who will benefit from therapy with such inhibitors. Future studies in animal models and analysis of clinical trials data with regard to BAD expression/phosphorylation are needed to determine the translational value of extensive efforts spent studying protein kinases that phosphorylate BAD.
